# The challenge of cystine and struvite stone formers: clinical, metabolic and surgical assessment

**DOI:** 10.1590/S1677-5538.IBJU.2015.0741

**Published:** 2016

**Authors:** Kleiton G. R. Yamaçake, Giovanni S. Marchini, Sabrina Reis, Alexandre Danilovic, Fábio C. Vicentini, Fábio C. M. Torricelli, Miguel Srougi, Eduardo Mazzucchi

**Affiliations:** 1 Seção de Endourologia, Divisão de Urologia do Hospital das Clínicas da Universidade de São Paulo Faculdade de Medicina de São Paulo, Brasil

**Keywords:** Urolithiasis, Urinary Tract Infections, Cystinuria, struvite [Supplementary Concept]

## Abstract

**Purpose:**

To compare the clinical, metabolic, and calculi characteristics of cystine and struvite stone patients after percutaneous nephrolithotripsy (PCNL).

**Material and Methods:**

Between January/2006-July/2013, 11 cystine stone patients were treated in our clinic. Of those, 3 were excluded due to incomplete follow-up. Eight cystine stone patients (2 with bilateral disease; 10 renal units-RU) were considered for further analysis. A cohort of 8 struvite stone formers (10RU) was matched having the same age, gender, body mass index (BMI) and Guys stone score. Analyzed parameters comprised demographic data, serum/urinary metabolic evaluation and surgical outcomes.

**Results:**

Both groups had 6 female patients. Groups were similar in regards to age, gender, BMI, stone burden, and serum creatinine (p=NS). All patients had PCNL as the first surgical treatment modality. Stone free rate (SFR) after the first PCNL tended to be lower (0%) in the cystine compared to the struvite group (40%)(p=0.08). Final SFR after secondary procedures increased to 70% in cystine and 80% in struvite patients (p=1.0); mean number of procedures to achieve stone free status was higher in the first group (3.57 vs. 2.0;p=0.028). Hypocitraturia was found in all patients, but struvite cases presented with lower mean urinary citrate levels (p=0.016). Other common abnormalities were elevated urinary pH (cystine 75% and struvite 62.5%;p=1.0) and low urinary volume (62.5%,37.5%;p=0.63).

**Conclusion:**

Multiple interventions and suboptimal stone free rates are trait of the significant stone burden of struvite and cystine patients. Underlying metabolic abnormalities characterized by increased urinary pH, hypocitraturia and low urinary volume are often encountered in both populations.

## INTRODUCTION

Cystinuria is the cause of 1–2% of all stones in adults and 6-8% of stones in the pediatric population (1). The prevalence has been estimated 1 in 1.000 to 1 in 17.000 in Europe and the United States, respectively (2). More than half of cystinuric patients eventually develop stone disease, regardless of their age, and three quarters of those present with bilateral disease (3). Definitive diagnosis is made by a 24-hour urine collection positive for cystine or stone analysis (4). The lifetime recurrence rate of cystine stone formers is extremely high. As a result, these individuals generally require multiple procedures for achieving a stone free status, with increased long-term risk for loss of renal function (4). In addition, some cystine stone patients have been shown to be present with chronic urinary tract infection, renal impairment and ultimately end-stage renal disease.

With a similar recurrent disease, struvite stone formers correspond to another challenging population with complex calculi and increased retreatment rates. To both these cohorts, percutaneous nephrolithotripsy (PCNL) is considered the treatment of choice if flexible ureteroscopic laser lithotripsy (URS) fails or significant stone burden imposes the primary procedure. In cystine stone patients, calculi hardness and multiplicity on several kidney sites commonly impose a challenging scenario, making any treatment approach difficult (5). The significant stone burden and associated infection are the main obstacles for accomplishing complete success when managing struvite stone formers. Besides, metabolic risk factors can occur in both cohorts and if not correctly addressed might lead to stone reformation and recurrence in a short period of time (6).

The personal impression that cystine stone formers are more difficult to manage surgically and metabolically than struvite cases is subjective. Therefore, we aimed to describe and compare the severity of cystine and struvite stone patients treated in a tertiary center in terms of clinical, metabolic, and stone surgical management characteristics.

## MATERIAL AND METHODS

### Matched Population

A retrospective search on our prospectively collected database was performed searching for patients with cystine stones treated at our tertiary center between January/2006 and July/2013. Eleven cystine stone patients were treated in our stone clinic and three were excluded due to incomplete follow-up. Eight cystine stone patients (two with bilateral disease; 10 renal units - RU) were considered for further analysis. For a matter of comparison, a matched cohort of infection stone formers was attained from the same database. We strictly chose patients with similar stone burden and clinical factors that might influence metabolic parameters in order to contrast the serum/urinary panel and surgical characteristic. A cohort of eight struvite stone formers (10RU) was matched having the same age, gender, body mass index (BMI) and Guys stone score. Stone volume was also calculated from measurements of non-contrast computed tomography (NCCT) by using the two largest axial diameters and the largest reconstructed coronal diameter. We used a previously validated formula to calculate the stone volume: V=length(cm)xwidth(cm)xdepth(cm) xπx0.167 (7). Analyzed parameters comprised demographic data, serum/urinary metabolic evaluation and surgical outcomes after PCNL.

### Surgical Evaluation

PCNL indications were renal stones >2cm, complex renal calculi, or smaller stones after failed shockwave lithotripsy (SWL). All procedures were performed under the supervision of an experienced surgeon. Patients underwent PCNL in the prone or supine position under general anesthesia according to the surgeon's preference.

All patients underwent a non-contrast computed tomography (NCCT) before surgery and in the first or second postoperative (PO) day. Preoperatively, all patients received a prophylactic third-generation cephalosporin at anesthesia induction or therapeutic antibiotics according to urine culture initiated 7 days before surgery. Patients with struvite stones had antibiotics started at least 24 hours before surgery even with negative urine culture results.

Success was defined as no clinically significant residual fragments (≤3mm) on PO NCCT. Stones were classified according to the Guy stone score (8, 9) and complications according to the Clavien modified grading system (10, 11).

### Stone Risk Assessment

The pre-operative metabolic evaluation included blood chemistry studies and two random 24-hour urine collections. Blood samples were taken from fasting patients in the morning and included the determination of serum calcium, sodium, potassium, uric acid, magnesium and creatinine (SCr) levels. Urinary volume, pH, calcium, uric acid, sodium, magnesium and citrate were assessed with 24-hour urine collection. Metabolic diagnoses were classified according to the institution laboratory parameters as follows: low volume (<2l/24h); increased pH (>6.5), hypercalciuria (>240mg/24h), hyperuricosuria (>750mg/24h in men; >650mg/24h in women), hypomagnesiuria (<60mg/24h), hypernatriuria (>200mEq/24h), and hypocitraturia (<290mg/24h). The metabolic evaluation was performed out of the period of active urinary infection in all patients.

### Statistical analysis

Statistical analysis was performed with SPSS version 20.0 (SPSS Inc., Chicago, IL). Results were expressed in proportion, mean, standard deviation and range. Groups were compared with Student T Test for numerical variables and Chi-Square or Fisher Exact Test for categorical variables. Significance was set at two-tailed p<0.05.

## RESULTS

### Demographic Data

Both groups were consisted of six female and two male patients. Groups were similar in regards to age, gender, BMI, Guys stone score, stone volume, initial and final serum creatinine and stone laterality (p=NS; Table-1). All patients underwent PCNL as the first surgical treatment modality.

**Table 1:** Demographic, clinical and surgical characteristics of cystine and struvite stone groups.

		Cystine Group (n=8; 10 RU)	Struvite Group (n=8; 10 RU)	
		Mean ± SD (range) / n (%)	Mean ± SD (range) / n (%)	p
Age (years)		24.75 ± 14.01 (9-52)	24.62 ± 13.08 (10-50)	0.98
Male / Female		2 (25%) / 6 (75%)	2 (25%) / 6 (75%)	1.0
BMI (kg/m^2^)		24.03 ± 3.81 (17.9 – 29)	24.00 ± 3.78 (16-28)	0.98
Initial Cr (mg/dL)		1.05 ± 0.65 (0.4-2.47)	0.98 ± 0.53 (0.57-1.81)	0.90
Final Cr (mg/dL)		1.32 ± 0.58 (0.77-2.5)	1.02 ± 0.51 (0.51-1.99)	0.32
Stone Volume (mm^3^)		6141.58 ± 5728.51 (755.49 – 21803.32)	7684.96 ± 6260.20 (572.33 – 16250.43)	0.59
Guys Stone Score				1.0
	1	0 (0%)	0 (0%)	
	2	3 (30%)	3 (30%)	
	3	6 (60%)	6 (60%)	
	4	1 (10%)	1 (10%)	
Side				0.57
	Right	5 (62.5%)	6 (75%)	
	Left	1 (12.5%)	0 (0%)	
Bilateral		2 (25%)	2 (25%)	
Stone free after 1st PCNL (RU)		0 (0%)	4 (40%)	0.08
Final stone free rate (RU)		7 (70%)	8 (80%)	1.0
N procedures for stone free (RU)		3.57 ± 1.04 (3-6)	2.0 ± 1.22 (1-4)	0.028
N procedures (RU)		3.0 (1-6) of 10 RU	2.2 (1-4) of 10 RU	0.19
Complications (Clavien)		0 (0%)	2 (Grade II, IIIb) (20%)	0.47
Stone composition: Pure / Mixed		2 (25%) / 6 (75%)	0 (0%) / 8 (100%)	0.46
Follow-up (months)		56.25 ± 24.90 (18-84)	39.0 ± 23.04 (12-84)	0.20

### Treatment Outcomes

Multiple procedures were a common finding in both groups: 7 out of 8 cystine patients had previous treatment (PCNL, SWL or open surgery), while 5 out of 8 struvite patients also had previous interventions. Overall, the number of procedures per RU was similar between groups: 3.0 (1–6) in the cystine versus 2.2 (1–4) in the struvite cohort (p=0.19).

**Figure 1:**
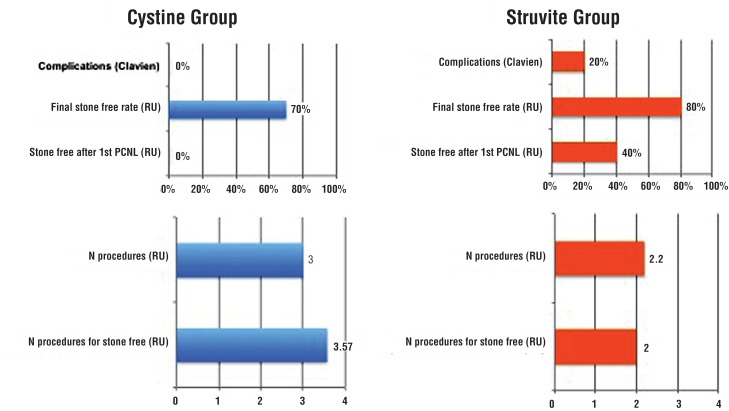
Surgical Outcomes - Struvite and Cystine Cohorts.

In the cystine group, no patient was rendered stone free with a single procedure (Figure-1). Two failures were treated with re-PCNL (two and three times), two with SWL (two and three times), four with URS (one patient twice), one with SWL plus URS and one with URS plus re-PCNL. Six of them became stone free (two after re-PCNL, one after SWL, one after URS, one after SWL and URS, one after URS and re-PCNL). Final stone free rate (SFR) was 70% and the mean number of procedures to achieve it was 3.6. Renal calculi consisted of pure cystine in two patients, a combination of cystine and calcium oxalate in four patients, and combination of cystine, uric acid and calcium oxalate in two patients.

In the struvite cohort, initial and final SFR were 40% and 80%, respectively (Figure-1). One initial failure was treated with re-PCNL (three times), four with SWL and one with ureterorenoscopy (URS). Four of them became stone free (three after SWL and one after re-PCNL). The mean number of procedures to achieve a SFR status was 2.0. No patient underwent bilateral PCNL simultaneously. All patients had mixed stone composition with ammonium magnesium phosphate and calcium oxalate or phosphate (Table-1).

Stone free rate (SFR) after the first PCNL tended to be lower (0/10RU) in the cystine compared to the struvite group (4/10RU) (p=0.08). Although the final SFR checked by NCCT after secondary procedures was similar between groups (7/10RU vs. 8/10RU; p=1.0), the mean number of procedures to achieve stone free status was significantly higher in the cystine cohort (p=0.028). Perioperative complications occurred only in struvite patients: one patient required blood transfusion after the PCNL (Clavien 2) and another patient had hemothorax requiring thoracoscopy (Clavien 3b). Both were treated uneventfully. One cystine patient developed a ureteral stricture 10 months after PCNL and required nephrectomy due to complete loss of ipsilateral renal function associated with pain.

### Metabolic Evaluation

Metabolic evaluation was carried out before the first surgical treatment at our Institution in all patients. During metabolic work-up, six out of eight patients in the cystine group were receiving captopril (angiotensin-converting enzyme inhibitor) and two out of eight were being treated with Thiola^®^ (Tiopronin). In the struvite group, all patients were being managed with prophylactic antibiotics during metabolic evaluation and none was receiving thiazides.

Serum analysis revealed increased creatinine levels in one (12.5%) patient in the cystine group and two (25%) patients in the struvite group (p=1.0). Hyperuricemia was also found in four (50%) cystine and one (12.5%) struvite patient (p=0.28). Absolute values were similar between groups and are presented on Table-2.

**Table 2:** Serum and urinary metabolic risk evaluation of cystine and struvite stone patients.

	Cystine Group (n=8; 10 RU)	Struvite Group (n=8; 10 RU)	
Serum Metabolic Panel	Mean ± SD (range)	Mean ± SD (range)	p
Creatinine (mg/dL)	1.05 ± 0.65 (0.4-2.47)	0.98 ± 0.53 (0.57-1.81)	0.601
Calcium (mg/dL)	9.57 ± 0.46 (8.8-10.2)	9.58 ± 0.50 (8.7-10.4)	0.959
Uric acid (mg/dL)	5.88 ± 1.37 (3.9-7.7)	5.012 ± 1.24 (2.8-7.3)	0.204
Magnesium (mg/dL)	1.85 ± 0.135 (1.79-2.1)	1.89 ± 0.137 (1.7-2.07)	0.556
Sodium (mg/dL)	139.63 ± 3.02 (135-143)	141.88 ± 3.137 (138-147)	0.166
24-hour Urinary Panel	Mean ± SD (range)	Mean ± SD (range)	p
pH	6.56 ± 0.52 (5.5-7)	6.31 ± 0.60 (5-7)	0.425
Volume (mL/24h)	2176.25 ± 869.07 (1500-3690)	2008.75 ± 454.54 (1100-2500)	0.637
Calcium (mg/24h)	60.43 ± 31.06 (30.8-120)	151.48 ± 92.86 (19.8-271.7)	0.130
Uric acid (g/24h)	0.39 ± 0.16 (0.18-0.62)	0.40 ± 0.16 (0.16-0.74)	0.857
Magnesium (mg/24h)	72.76 ± 35.66 (29.12-132.51)	69.74 ± 33.87 (18.54-123.99)	0.870
Sodium (meq/24h)	155 ± 48.74 (54-198)	162.75 ± 18.18 (139-194)	0.680
Citrate (mg/24h)	295.81 ± 106.61 (176-471)	139.89 ± 119 (26.6-314.6)	0.016

**Figure 2:**
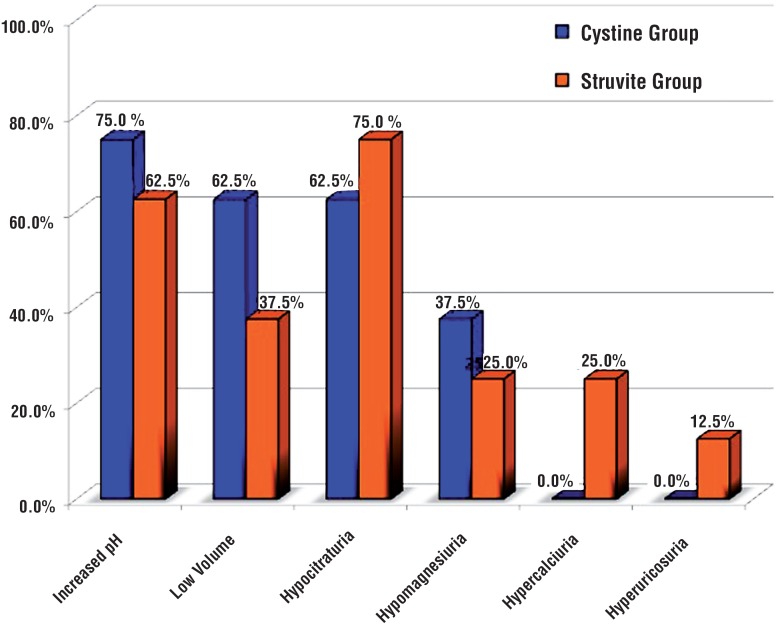
Urinary Stone Risk - Struvite and Cystine Patients

On urinary metabolic evaluation, most patients of both groups showed significant lithogenic urinary abnormalities (Figure-2). Hypocitraturia occurred in 75% of patients in the struvite cohort and 62.5% in the cystine group, with lower mean urinary citrate levels in the struvite cohort (p=0.016; Table-2). Increased pH and low urinary volume were found in 62.5% and 37.5% in the struvite cohort and in 75% and 62.5% of patients in the cystine group (p non-significant). Low magnesium was found in 25% and 37.5% of struvite and cystine patients. In the struvite group, hypercalciuria and hyperuricosuria were seen in 25% and 12.5% of patients, respectively.

## DISCUSSION

More than half of cystinuric patients will develop cystine stones during their lifetime, with a high incidence of bilateral stone formation (12, 13). In our series, most cystine and struvite patients had right-sided stones. However, bilateral disease was seen in 25% of cases in both groups. Most cystine patients were young and not obese, which is in accordance to the literature (12). The management of cystine stone disease is an important clinical issue because of its recurrent nature. Particularly, genetic factors play more important role in the recurrence and regrowth rates for cystine stone disease, although the etiology of urolithiasis is influenced by socio-economic, geographical and genetic factors (12).

To our knowledge, there is no report comparing the metabolic profile and surgical outcomes in cystine and struvite stone patients. We used the Guys stone score to match the severity of stone burden from the cystine group with a struvite cohort. In the present study, the stone free rate was found to be 0% for cystine and 40% for struvite patients after the first PCNL. However, this rate increased to 70% and 80%, respectively, with additional procedures.

A recent study evaluated ten cystine patients with mean stone diameter of 11.2mm (5-30mm) submitted to ureterorenoscopy (14). Complete stone clearance was achieved in 15 out of 21 procedures (71%). In five cases (24%), significant residual fragments were found and in the one case (5%) the procedure was ineffective. After secondary procedures, complete stone clearance was achieved in five more cases, with final stone free rate of 95%. These results are superior to ours, probably due to the severity of our patients who underwent PCNL. In selected cases, especially if early diagnosis is possible, minimally invasive procedures allow excellent outcomes, improving patient quality of life while reducing morbidity. In another study evaluating 65RU in 51 children with large cystine stones (mean burden of 3.3cm^2^) treated with PCNL, the stone free rate was 63.1% (41/65RUs) (15). After additional endoscopic procedures the final stone-free rate was increased to 73.8%. Importantly, the recurrence rate for children who once became stone free was as high as 31.2%, while re-growth rate for children with residual fragments was 29.4%.

In a similar perspective, Shen et al. performed a comparison of renal function and metabolic risk factors in 30 cystine stone and 30 calcium oxalate stone formers (16). The authors revealed a mean number of procedures per cystine stone former of 4.7±5.7/patient, which was higher than that of calcium oxalate stone formers (2.0±1.4/patient). Calcium oxalate stones are usually not as complex as struvite stones, with lower recurrence rates. We showed that cystine patients might require even more procedures than the severe cases of infection calculi to achieve a stone free status. In the same series, metabolic abnormalities could be demonstrated in 80% of the cystine and in 100% of the calcium oxalate stone patients (16). The most common metabolic abnormalities in cystine and calcium oxalate stone patients were hypercalciuria (46.7%) and hypocitraturia (80%), respectively. In our study, 62.5% of cystine stone patients presented with hypocitraturia and none with hypercalciuria. Moreover, they found that cystine stone patients had higher urinary citrate and lower urinary oxalate and creatinine than calcium oxalate stone patients. We found lower values of urinary citrate in the struvite cohort. Unfortunately, oxalate could not be measured as a routine for our patients. Sakhaee and colleagues evaluated 27 cystine stone patients and identified hypercalciuria in 19%, hyperuricosuria in 22%, and hypocitraturia in 44% of patients (6). Differences in diet, nutritional habits and medical treatment might explain those discrepancies.

The main goal when treating patients with cystinuria is to prevent renal stone formation, which may be accomplished by reducing the urinary concentration of cystine with high fluid intake and alkalinization. Total urinary volume should reach four liters per day and dietary recommendations should include a low methionine diet by avoiding animal proteins. Finally, sodium intake should be reduced and fiber intake increased (17). Medications like D-penicillamine, alpha-mercaptopropionylglycine, angiotensin-converting enzyme inhibitor, or bucillamine may be used. Alpha-mercaptopropionylglycine has been reported to prevent stone formation in 85% of patients but the incidence of adverse effects is not negligible (18, 19). Most of our cystine patients were receiving captopril due to hypertension and cystine stone formation. The captopril-cystine complex formed as a result of its thiol group binding to cystine is reported to increase cystine solubility by about 200 times. Although the efficacy of captopril is still in question, it is recommended as the drug of choice by Cohen et al. in patients with hypertension (20, 21). Cystine patients should also be followed closely. Renal ultrasound and an aggressive treatment policy are advocated in order to avoid rapid silent stone grown. Retrograde endoscopic surgery using flexible ureteroscopy is gaining in popularity and might potentially diminish the necessity of PCNL in such patients.

The finding of urinary abnormalities in all cystine stone formers reinforces the importance of medical treatment for those patients. Although most cystine patients had already undergone aggressive surgical treatment, 75% had lower urinary volume at initial metabolic evaluation and only two patients achieved the desired 3-4 liters urinary output per day. Inadequate previous medical counseling or low compliance with recommendations might play a role and contribute to the high stone recurrence rate in the young cystine cohort. Those patients should be managed in excellence centers where surgical and medical management go hand in hand in a multidisciplinary approach.

In addition, the management of struvite stones is also demanding. According to the 2005 American Urological Association guideline on management of staghorn calculi, surgical removal of stone material is the standard therapy and the main goal is its complete removal (21). Moreover, culture-specific preoperative and perioperative antibiotics are critical to prevent sepsis during the surgery. Long-term, low dose culture-specific antimicrobials are important to prevent new stone growth and progression after surgery. Also by minimizing urease concentrations, small fragments after surgery may be eradicated (22). Is resemblance of the mentioned for cystine patients, early reevaluation with renal ultrasound and NCCT when necessary are essential to avoid asymptomatic stone regrowth. Flexible ureteroscopy has become an excellent tool to increase the stone free rate in struvite patients who underwent PCNL and have small residual fragments. The same surgical technique might be applied if stone recurrence is detected early and stone burden is not large.

Although this is the first study to compare in a matched cohort fashion cystine and struvite patients in terms of surgical outcomes and metabolic risk factors, it is not without limitations. The data were verified retrospectively, susceptible to selection and measurement bias. We aimed to decrease those effects by doing a matched study with strict criteria for comparison and we only selected cases with complete data. Another limitation is the small number of patients enrolled and this is a result of the rarity of cystinuric patients worldwide as in our community. We also should consider the possible impact of medications on our metabolic assessment. In similarity to other rare and severe illnesses, a multi-institutional study combining data and experience from diverse centers worldwide would allow gathering of a more substantial number of cystine patients and provide better understanding the disease.

## CONCLUSIONS

Patients with cystinuria and struvite calculi require repeated surgical interventions to reach suboptimal success rates. Nevertheless, with recent advances in surgical techniques, those outcomes might be accomplished with minimally invasive procedures. Hypocitraturia, low urinary volume and high pH are the most common findings in both groups of patients other than elevated urinary cystine levels and urinary infection.
